# The Influence of Bisphenol A (BPA) on Neuregulin 1-Like Immunoreactive Nerve Fibers in the Wall of Porcine Uterus

**DOI:** 10.3390/ijms19102962

**Published:** 2018-09-28

**Authors:** Liliana Rytel

**Affiliations:** Department of Internal Disease with Clinic, Faculty of Veterinary Medicine, University of Warmia and Mazury, ul. Oczapowskiego 14, 10-719 Olsztyn, Poland; liliana.rytel@gmail.com

**Keywords:** bisphenol A, uterus, neurotransmitter, Neuregulin-1, pig

## Abstract

Bisphenol A (BPA), a substance commonly used in the manufacture of plastics, shows multidirectional negative effects on humans and animals. Due to similarities to estrogens, BPA initially leads to disorders in the reproductive system. On the other hand, it is known that neuregulin 1 (NRG-1) is an active substance which enhances the survivability of cells, inhibits apoptosis, and protects tissues against damaging factors. Because the influence of BPA on the nervous system has also been described, the aim of the present study was to investigate for the first time the influence of various doses of BPA on neuregulin 1-like immunoreactive (NRG-1-LI) nerves located in the porcine uterus using the routine single- and double-immunofluorescence technique. The obtained results have shown that BPA increases the number and affects the neurochemical characterization of NRG-1-LI in the uterus, and changes are visible even under the impact of small doses of this toxin. The character of observed changes depended on the dose of BPA and the part of the uterus studied. These observations suggest that NRG-1 in nerves supplying the uterus may play roles in adaptive and protective mechanisms under the impact of BPA.

## 1. Introduction

Neuregulins are members of epidermal growth factor family proteins, which play essential roles in embryogenesis and allow normal development of many internal organs and systems, including the nervous system and heart. Until now, four types of neuregulins have been discovered [1]: (1) Neuregulin-1 occurring in various isoforms, playing a wide range functions in the living organism, first of all in the development of the nervous system and heart; (2) Neuregulin 2—inducing mainly the growth and development of epithelial, neuronal, and glial cells; (3) Neuregulin 3, which may act on receptor tyrosine-protein kinase ERBB-4 and is involved in pathological processes connected with schizophrenia and Hirschsprung’s disease; and (4) neuregulin 4, which is mainly expressed and secreted by brown adipocytes and may take participation in mechanisms during epithelial cell-related diseases, tumors, and glycolipid metabolic diseases. The best known member of the neuregulin family is neuregulin 1 (NRG-1). This substance, described for the first time in 1992 [2], is a 44-kD glycoprotein, which contains amino acid sequence typical for epidermal growth factor and therefore may interact with the ERBB receptors [3]. Stimulation of these receptors causes an increase in the survivability of cells, angiogenesis, and inhibition of apoptosis [4]. Until now, the presence of NRG-1 has been reported in various parts of the nervous system. First of all, this substance has been observed in the central nervous system [5], where it is responsible for survivability and differentiation of oligodendrocytes [6], processes connected with memory, learning, and development of cerebral cortex [7], as well as the control of sexual maturation [5]. In turn, regarding the peripheral nervous system [5], NRG-1 has been observed in dorsal root ganglia [8], neuronal cells, and nerves in the digestive tract [9]. The functions of this substance within the peripheral nervous system are not fully explained, but it is known that NRG-1 could expect to show neuroprotective effects by the influence on survivability and regeneration of Schwann cells [10]. This substance is also involved in regulatory processes connected with the activity of neuromediators and/or neuromodulators, receptors, and neuronal ion channels [5].

In spite of a relatively large number of studies on the presence of NRG-1 in various internal tissues and organs [4,5,6,7], the knowledge concerning distribution and functions of this substance within the female reproductive system is extremely scanty. Previous studies have described the presence of NRG-1 in the ovary under physiological conditions and during cancer [11], as well as in different parts of the uterine wall, including luminal epithelium, superficial, and deep uterine glands, stroma, and smooth muscles [12]. Moreover, functions of NRG-1 within the female reproductive system still remain not fully explained. It is known that in the ovary, the described substance is involved in regulatory processes connected with the survival and differentiation of granulosa cells, as well as oocyte maturation [13]. In turn, within the uterus NRG-1 plays roles in endometrium maturation [14] and estrus cycle regulation [15]. Furthermore, important functions of NRG-1 during pregnancy have been confirmed. Namely, NRG-1 is not only involved in implantation [16], as well as in endometrial cell proliferation and differentiation during gestation, but probably also in the communication between the embryo/fetus and uterus by the placenta [12]. Some previous studies have described participation of NRG-1 signaling during pathological states of the uterus, including endometrial cancer [14].

It should be pointed out that until now the presence of NRG-1 in the nerve fibers supplying the uterine wall has not been studied, although previous investigations have described a wide range of active neuronal factors located in neuronal cells and fibers innervating this organ [16]. So the aim of the present investigation was to investigate the distribution and neurochemical characterization of the nerves immunoreactive to NRG-1 within body and horns of the porcine uterus in physiological conditions and under the influence of bisphenol A (BPA), which is one of the most widespread toxins in the environment [17]. This is because BPA is commonly used in the production of plastics and it is a component of various everyday objects, such as bottles, food containers and cans, paints, thermal paper, elements of cars and furniture, household goods, and many other things. BPA can flush out from the above mentioned things, penetrate into the food, water, soil, and air, and then get into living organisms by the gastrointestinal tract, skin, or lungs [18]. Due to the similarity to estrogen and the possibility of estrogen receptor stimulation, BPA is identified as an endocrine disruptor [19]. Previous studies have shown that BPA may have a negative influence on various internal systems and organs. First of all, it affects the reproductive system, in which it causes changes in the uterine mucosal layer, cell apoptosis, and exposure of estrogen receptors [20]. Excessive exposure to BPA also results in increased risk for development of polyps, endometriosis, and cervical cancer [21], as well as leading to disturbances during pregnancy and anomalies in the fetus [22].

The second system which is very sensitive to impact of BPA is the nervous system. It is known that BPA has neurotoxicity effects [23]. Administration of this substance results in disturbances in the development of dendrites and synapses [24], as well as changes in the conduction of pain and sensory stimuli [25]. The above mentioned processes are probably connected with changes in the levels of neuronal factors responsible for the growth and differentiation of neurons, and fluctuations in the intraneuronal calcium concentration [26]. Previous studies have shown that even single doses of BPA may result in changes in functions of the brain, manifesting in disorders in behavior and memory [27]. Contrary to the central nervous system, the impact of BPA on the peripheral nervous system is not fully explained.

These effects are probably connected with BPA-induced changes in the calcium concentration and active substances responsible for growth and differentiation of neuronal cells, as well as stimuli conduction both in the central and peripheral nervous system [28]. The above mentioned mechanisms mean that even single exposure to BPA in the periparturient period results in disorders in higher-level brain functions including behavior, learning, and memory [29].

Indeed, the reproductive and nervous systems are not the only parts of the living organism on which BPA has a negative influence. It is known that this substance may cause disorders in the immunological, endocrine, respiratory and excretory systems, digestive tract and liver, lungs, heart, and blood vessels [30]. Previous studies have shown a link between excessive exposure to BPA and various civilization diseases, including diabetes, hypertension, heart attack, or neoplasms [31]. Moreover, it is known that BPA changes the neurochemical characterization of neurons and nerves within the peripheral nervous system in various internal organ including intestine, heart, and liver [32,33,34,35], but until now the participation of NRG-1 in mechanisms connected with BPA intoxication has not been studied.

The using of the domestic pigs in the present experiment has not been accidental. Due to relatively well-known analogies in terms of physiology, biochemistry, and first of all organization and neurochemical characterization of the nervous system between humans and domestic pigs [36], this animal species is increasingly used as an animal model to study the pathological processes taking part in the human organism [37]. Hence, the results obtained during the present study may be the first step to establish of roles of NRG-1 within uterine innervation in intoxication with BPA in humans.

## 2. Results

During the present study, no clinical symptoms of the treatment with BPA were observed both under the impact of low and high doses of the toxin. However, the influence of BPA on the neurochemical characteristics of nerve fibers was visible.

### 2.1. The Influence of BPA on the Number of NRG-1-Positive Nerves

During this investigation, the presence of NRG-1 was observed in nerve fibers located in the mucosal and muscular layers of the uterine corpus and horns both in physiological conditions and under the impact of BPA. NRG-1-positive fibers were visible and thick, but their numbers were not high (Table 1 and Table 2). In control animals, the average number of intramuscular nerves immunoreactive to NRG-1 per observation field amounted to 5.45 ± 0.52 in the corpus of the uterus, and 3.89 ± 0.38 and 3.78 ± 0.34 in the right and left uterine horns, respectively. In the mucosal layer, the number of neuregulin 1-like immunoreactive (NRG-1-LI) fibers was similar: In the uterine corpus—5.08 ± 0.43, in the right horn—2.93 ± 0.45, and in the left horn—3.08 ± 0.32. Both low and high doses of BPA increased the number of NRG-1-positive nerves located in the muscular layer in all parts of the uterus studied, and the most visible changes were observed under high doses of the toxin. Within the uterine corpus, the average number of NRG-1-LI nerves amounted to 8.38 ± 0.43 under low doses of BPA, and 12.29 ± 0.58 in animals receiving high doses of the toxin. The number of nerves immunoreactive to NRG-1 in the muscular layer of the uterine horns was smaller. In pigs in which small doses of BPA were administered, these values were 5.38 ± 0.35 in the right horn and 5.18 ± 0.35 in the left horn. In turn, under the high dose of BPA, the number of NRG-1-LI nerves in the right horn amounted to 9.20 ± 0.46, and in the left horn 9.97 ± 0.37. The changes caused by BPA were less visible in the case of NRG-1-LI nerves located in the mucosal layer. Contrary to the muscular layer, low doses of BPA did not cause changes in the number of nerves immunoreactive to NRG-1. The number of such nerves in animals of E1 group achieved 4.78 ± 0.43 in the uterine corpus, 3.19 ± 0.31 in the right horn, and 3.47 in the left horn. These values were not statistically significantly different from those observed in control animals. In turn, high doses of BPA increased the number of NRG-1-LI nerves located in the mucosal layer to 7.86 ± 0.29 in the uterine corpus, 5.70 ± 0.54 in the right horn, and 6.25 ± 0.45 in the left horn. The impact of BPA did not cause the morphology of NRG-1-LI nerves to change. Under influence of both doses of the toxin, these nerves (like in the control animals) were visible, thick, and formed bundles.

### 2.2. BPA-Induced Changes in the Neurochemical Characterization of NRG-1-Positive Nerves in the Muscular Layer of the Uterine Corpus

**Figure 1:**
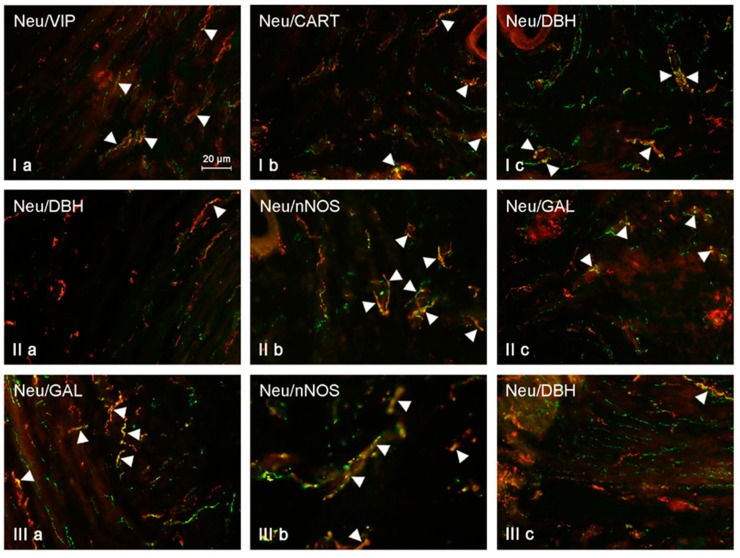
Neuregulin (NRG)-positive nerves (NRG-1+ green) immunoeractive to dopamine beta-hydroxylase (DBH), substance P (SP), vasoactive intestinal polypeptide (VIP), neuronal isoform of nitric oxide synthase (nNOS), galanin (GAL), and cocaine- and amphetamine-regulated transcript (CART) (red) in the muscular layer of the uterine body of control animals (**a**) and pigs treated with low (**b**) and high (**c**) dose of bisphenol A. Nerves simultaneously immunoreactive to NRG-1 and other substances (yellow) are indicated with arrowheads.

During the present study, a wide range of neuronal active substances was noted in the nerves immunoreactive to NRG-1 (Table 1, Figure 1). Under physiological conditions in the muscular layer, the greatest percentage of NRG-1-LI nerves located in the uterine corpus simultaneously showed presence of SP (34.37 ± 1.63% of all NRG-1-LI nerves) and VIP (31.79 ± 1.21%). A slightly lower percentage of NRG-1-positive fibers was also immunoreactive to CART (24.60 ± 1.15%) and GAL (14.59 ± 0.97%). The lowest number of nerves immunoreactive to NRG-1 showed the presence of DBH. The co-localization of these substances was observed only in single fibers (0.34 ± 0.06% of all NRG-1-LI nerves). Low doses of BPA caused an increase in the percentage of NRG-1-LI nerves simultaneously immunoreactive to the majority of neuronal factors studied. In the muscular layer of the uterine corpus of animals from the E1 group, the largest number of NRG-1-LI nerves showed the presence of VIP. The percentage of such nerves was 42.28 ± 1.17% of all NRG-1-positive fibers (an increase by about 11 percentage points (pp.) in comparison to control animals). SP was noted in 38.14 ± 2.05% of all NRG-1-LI nerves, and differences in the percentage of NRG-1+SP+ nerves between control animals and pigs under the impact of low doses of BPA were not statistically significant. In turn, low doses of the toxin caused an increase in the percentage of NRG-1+/GAL+ fibers (to 31.38 ± 1.75%, by about 17 pp.) and nerves simultaneously immunoreactive to NRG-1 and CART (to 30.35 ± 1.46%, by about 6 pp.). A very clear increase was also noted in the case of NRG-1+/DBH+ nerves. The percentage of these fibers under low doses of BPA amounted to 3.45 ± 0.30% and was about ten times higher than that observed in control animals. Changes observed in the neurochemical characterization of NRG-1-LI nerves in the muscular layer of the uterine corpus under the impact of high doses of BPA were more visible. In animals of E2 group, the largest number of these nerves was also immunoreactive to VIP (49.24 ± 1.25%, an increase of about 18 pp.). Slightly less number of NRG-1-LI nerves showed the presence of CART (42.49 ± 1.48%, an increase of about 18 pp.) and SP (39.03 ± 1.73%, an increase of about 5 pp.). Similarly to low dose of BPA, high dose of this toxins also did not cause the statistically significant changes in the number of NRG-1+/SP+ nerves. In the muscular layer of the uterine corpus of animals from the E2 group, GAL was noted in 30.58 ± 1.69% of all NRG-1-LI fibers (increase by about 16 pp. in comparison to the control animals). In turn, the presence of DHB was observed in 8.31 ± 0.39% of nerves immunoreactive to NRG-1, and this value was above twenty times higher than that observed under physiological conditions. 

### 2.3. BPA-Induced Changes in the Neurochemical Characterization of NRG-1-Positive Nerves in the Muscular Layer of the Uterine Horns

**Figure 2:**
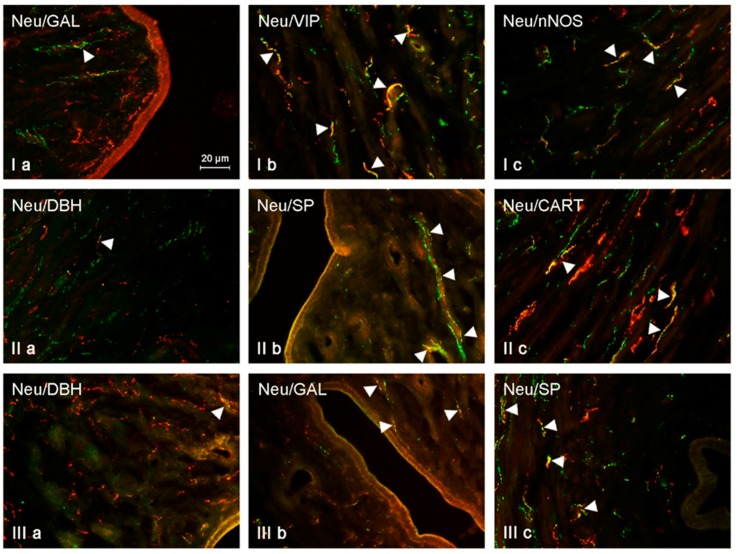
NRG-positive nerves (NRG-1+ green) immunoeractive to DBH, SP, VIP, nNOS, GAL, and CART (red) in the mucosal layer of the uterine body of control animals (**a**) and pigs treated with low (**b**) and high (**c**) dose of bisphenol A. Nerves simultaneously immunoreactive to NRG-1 and other substances (yellow) are indicated with arrowheads.

Neurochemical characterization of NRG-1-positive nerves in the muscular layer of the uterine horns was slightly different from that observed in the corpus of the uterus (Table 1, Figure 2). In this part of the uterus under physiological conditions, the largest number of NRG-1-LI nerves was also immunoreactive to SP (29.15 ± 2.12% of all NRG-1-LI nerves in the right horn and 28.97 ± 1.28% in the left horn) and VIP (26.02 ± 1.13% in the right horn and 26.67 ± 2.20% in the left horn). A slightly lower number of NRG-1-positive nerves showed the presence of CART (19.02 ± 1.08% in the right horn and 18.60 ± 1.72% in the left horn) and GAL (10.15 ± 0.85% in the right horn and 9.62 ± 0.89% in the left horn). Contrary to the above mentioned substances, which are noted in smaller number of NRG-1-LI intramuscular nerves in the uterine horns in the comparison to the corpus of the uterus, NRG-1+/DBH+ fibers in the horns were more numerous than in the corpus. The percentage of such fibers amounted to 2.77 ± 0.24% of all fibers immunoreactive to NRG-1 in the right horn and 2.85 ± 0.27% in the left horn. Low doses of BPA did not influence on the degree of co-localization of NRG-1 with SP or VIP in the intramuscular nerves within the uterine horns. In animals of the E1 group, the percentage of NRG-1+ nerves, which were also immunoreactive to SP, was 32.02 ± 0.94% in the right horn and 32.52 ± 1.65% in the left horn, and the percentage of NRG-1+/VIP+ nerves was 29.74 ± 1.55% and 29.04 ± 1.55%. These values were not statistically significantly differ from those observed in control animals. Low doses of BPA caused an increase in the number of all other populations of nerves studied. The presence of CART was noted in 26.45 ± 1.63% of all NRG-1-LI nerves in the right horn and in 25.12 ± 1.27% in the left horn (in both horns the increase amounted to about 7 pp.). Low doses of BPA also affect the number of NRG-1+/GAL+ nerves. The percentage of these nerves amounted to 25.75 ± 1.18% in the right uterine horn (increase by about 15 pp. in comparison to the control animals) and 27.43 ± 1.67% in the left horn (increase of about 18 pp.). Clear influence of low doses of BPA was also visible in the case of NRG-1+/DBH+ fibers. Their number increased to 10.45 ± 0.44% in the right horn and 8.89 ± 0.41% in the left horn (in both horns about four-fold increase in comparison to control animals). Generally, changes caused by high doses of BPA observed in neurochemical characterization of intramuscular NRG-1-LI in the uterine horns were more visible. The largest number of NRG-1-LI nerves was simultaneously immunoreactive to CART (39.34 ± 1.93% in the right horn and 39.23 ± 1.78% in the left horn, an increase of about 13 pp. in comparison to the control animals). A somewhat lower percentage of NRG-1-positive nerves showed the presence of SP (34.50 ± 1.38% in the right horn and 34.35 ± 1.21% in the left, an increase in comparison to control animals of about 5 pp.) and VIP (33.85 ± 1.20% in the right horn and 33.42 ± 1.21% in the left horn, increase of about 7 pp.). High doses of BPA also caused a visible increase in the number of NRG-1+/DBH+ nerves. The percentage of such nerves amounted to 11.69 ± 0.12% in the right horn and 11.26 ± 0.20 in the left horn (increase of about 8 pp.). Interestingly, the number of NRG-1+/GAL+ nerves after administration of high doses of BPA (19.58 ± 1.02% in the right horn and 20.49 ± 1.36% in the left horn, increase of about 10 pp.) was admittedly higher than in control animals, but clearly lower than that observed under the impact of a low dose of BPA.

### 2.4. BPA-Induced Changes in the Neurochemical Characterization of NRG-1-Positive Nerves in the Mucosal Layer of the Uterine Corpus

**Figure 3:**
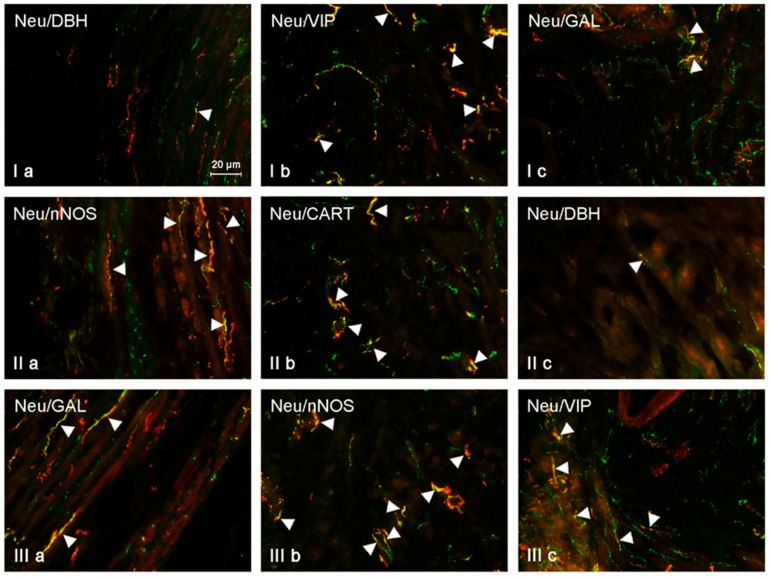
NRG-positive nerves (NRG-1+ green) immunoeractive to DBH, SP, VIP, nNOS, GAL, and CART (red) in the muscular layer of the uterine horns of control animals (**a**) and pigs treated with low (**b**) and high (**c**) dose of bisphenol A. Nerves simultaneously immunoreactive to NRG-1 and other substances (yellow) are indicated with arrowheads.

The neurochemical characterization of NRG-1-LI nerves located in the uterine mucosa was slightly different from that observed in the muscular layer (Table 2, Figure 3). Under physiological conditions, the largest number of NRG-1-positive fibers located in the mucosa of the uterine corpus was also immunoreactive to SP (31.55 ± 0.92%) and VIP (24.10 ± 0.98%). A slightly lower number of NRG-1-LI nerves simultaneously contained CART (18.56 ± 1.39%) and GAL (10.52 ± 0.47%). The presence of DBH was observed (like in the muscular layer) only in single NRG-positive fibers (1.87 ± 0.41% of all NRG+ nerves). Low doses of BPA caused an increase in the degree of co-localization of NRG-1 with almost all neuronal factors studied in the intramucosal nerves within the uterine mucosal layer. The only exception concerned NRG-1+/SP+ fibers. Their number in animals of E1 group amounted to 34.25 ± 0.76%, and this value was not statistically significantly differ from that observed in control pigs. The largest number of NRG-1-LI nerves in the mucosal layer of the uterine corpus in animals receiving low doses of BPA contained VIP (35.53 ± 0.69%, increase of about 11 pp.). Low doses of BPA also caused an increase in the percentage of NRG+/GAL+ nerves (to 25.86 ± 1.04%, an increase of about 15 pp.) and NRG+/CART+ fibers (to 22.61 ± 0.82%, an increase of about 4 pp.). A ten-fold increase was observed in the case of nerves simultaneously immunoreactive to NRG-1 and DBH, the percentage of which in animals treated with small doses of BPA amounted to 10.73 ± 0.57%. In animals receiving high doses of BPA, the largest percentage of NRG-1-LI nerves located in the mucosa of the uterine corpus showed the presence of nNOS (45.33 ± 1.99%, an increase of about 23 pp. in comparison to the control group), VIP (41.44 ± 0.97%, an increase of about 17 pp.) and/or CART (36.36 ± 1.67%, an increase of about 18 pp.). SP was noted in 34.05 ± 1.75% of all NRG-1-positive fibers, and this value was not statistically significantly different from those observed in control animals and pigs receiving low doses of BPA. In turn, GAL was observed in 25.27 ± 0.70% of NRG-1-LI nerves. This value was admittedly higher than that noted in the control group (by about 15 pp.), but was similar to that observed in animals receiving low doses of BPA. Moreover, high doses of BPA caused a clear increase in the number of NRG-1+/DBH+ nerves, the percentage of which amounted to 17.35 ± 1.08% of all NRG-1-LI nerves and was about 17 times higher than in the control group.

### 2.5. BPA-Induced Changes in the Neurochemical Characterization of NRG-1-Positive Nerves in the Mucosal Layer of the Uterine Horns

**Figure 4:**
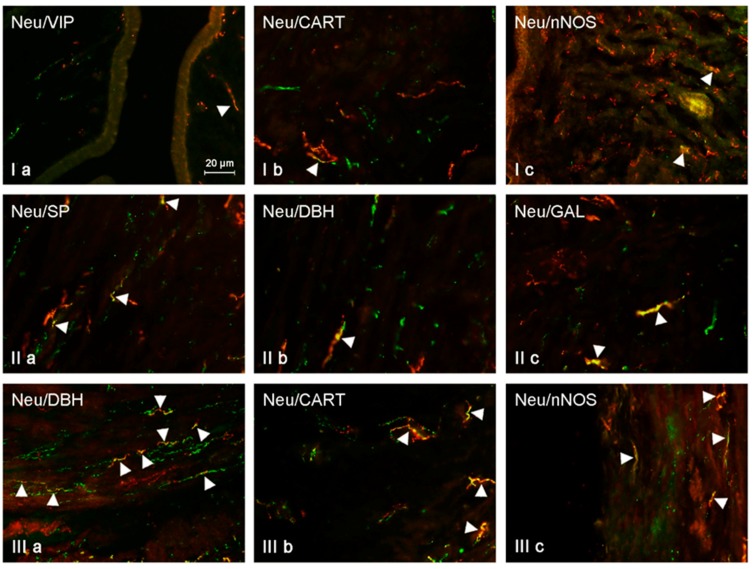
NRG-positive nerves (NRG-1+ green) immunoeractive to DBH, SP, VIP, nNOS, GAL, and CART (red) in the mucosal layer of the uterine horns of control animals (**a**) and pigs treated with low (**b**) and high (**c**) dose of bisphenol A. Nerves simultaneously immunoreactive to NRG-1 and other substances (yellow) are indicated with arrowheads.

Under physiological conditions, the largest number of NRG-1-positive nerves located in the mucosal layer of the uterine horns contained SP (30.45 ± 0.78% in the right horn and 30.72 ± 1.15% in the left horn) and/or VIP (21.02 ± 1.46% and 21.96 ± 1.03%, respectively) (Table 2, Figure 4). In the case of nerves simultaneously immunoreactive to NRG-1 and CART, these values amounted to 14.77 ± 1.13% and 14.30 ± 1.08%, respectively (Table 2, Figure 4). The smallest number of NRG-1-positive fibers showed the presence of GAL (7.99 ± 0.77% in the right horn and 8.15 ± 0.79% in the left horn) and/or DBH (7.32 ± 0.62% and 7.26 ± 0.99%, respectively). The administration of low doses of BPA resulted in an increase in the percentage of NRG-1-positive nerves simultaneously immunoreactive to SP, GAL, and DBH. The presence of SP was noted in 35.45 ± 0.78% of NRG-1+ nerves in the right horn (an increase of about 5 pp. in comparison to control animals) and in 34.89 ± 1.72% in the left horn (an increase of about 4 pp.). In the case of NRG-1+/GAL+ nerves, these values achieved 21.76 ± 0.7% and 21.58 ± 1.24%, respectively, and were higher than those observed in the control group by about 13 pp. A similar increase (about 13 pp.) was also observed in the case of NRG-1+/DBH+ nerves, the number of which amounted to 19.97 ± 0.87% in the right horn and 20.71 ± 1.36% in the left horn. Low doses of BPA did not affect the degree of co-localization of NRG-1 with CART and/or VIP. The percentage of the first mentioned nerves amounted to 18.86 ± 1.25% in the right horn and 18.46 ± 1.85% in the left horn. In turn, the percentage of NRG-1+/VIP+ nerves was 24.83 ± 0.95% and 24.51 ± 1.93%, respectively. In both of these cases, values noted in the E1 group were not statistically significantly different from those observed in the control pigs. High doses of BPA caused an increase in the number of nerves in which NRG-1 co-localized with CART, GAL, and/or DBH. The largest number of NRG-1-LI nerves in the mucosal layer of the uterine horns in animals treated with high doses of BPA showed the presence of CART (36.35 ± 0.99% in the right horn and 35.59 ± 1.43% in the left horn, an increase in comparison to control animals of about 22 pp.). Clearly visible changes also concerned NRG-1+/DBH+ fibers. Their percentage amounted to 38.62 ± 1.73% of all NRG-1-LI nerves in the right horn and 39.96 ± 1.06% in the left horn (in both horns an increase of about 19 pp. in comparison to the control group was achieved). The percentage of NRG-1+/GAL+ nerves in animals under the impact of high doses of BPA amounted to 20.00 ± 1.17% in the right horn and 19.84 ± 1.06% in the left horn. These values were higher than in the control animals (by above 10 pp.), but they were similar to those observed in animals after the administration of low doses of BPA. High doses of BPA did not affect the number of NRG-1+/VIP+ nerves. Their percentage amounted to 24.50 ± 1.24% in the right horn and 23.74 ± 1.03% in the left horn, and these values were not statistically significantly different from those observed under physiological conditions. A similar situation was observed in the case of NRG-1+/SP+ fibers. In the animals of E2 group, the percentage of such fibers achieved 32. 24 ± 1.07% in the right horn and 33.02 ± 1.30% in the left horn. These values were similar to those observed in the control group and, interestingly, they were lower than the number of NRG-1+/SP+ nerves noted in animals treated with low doses of BPA. All results obtained during the present study are summarized in Table 1 and Table 2.

## 3. Discussion

The results obtained during the present study clearly show that NRG-1 is present in nerves within various parts of the porcine uterus. It is the first report concerning the occurrence of NRG-1 in the innervation of the reproductive system. Previous studies have described this substance in the ovary and uterine luminal epithelium, superficial and deep glands, stroma, and smooth muscles [38,39]. The presence of nerves immunoreactive to NRG-1 observed during the present study may suggest that this substance plays important roles in the innervation of the uterus as a neuromediator and/or neuromodulator. In turn, a wide range of other neuronal factors observed in NRG-1-LI nerves may indicate multidirectional roles of this substance. However, in spite of the fact that the participation of NRG-1 in the control of maturation and regulation of the estrus cycle have been confirmed [15], the exact roles of these substance in neuronal control of the reproductive system are pure conjecture. It can be concluded that these roles are similar to those which have been investigated in other parts of the nervous system. Previous studies have described that in the central nervous system NRG-1 affects oligodendrocytes, supporting development, differentiation, and survival of these cells [40]. In the brain, NRG-1 also participates in processes taking place in the cortex affecting higher-level brain functions, including memory and learning, as well as development of cortical neuronal cells [41].

The knowledge concerning roles of NRG-1 in the peripheral nervous system is even more limited. It is known that NRG-1 influences Schwann cells, showing neuroprotective and regenerative effects [42], and may change the levels and activity of other neuronal active substances (mainly neuromediators and/or neuromodulators) and their receptors [43]. Some previous studies have shown the participation of NRG-1 in processes regulating the activity of ion channels within neuronal cells [44].

One of the ways to better understand the functions of less-known neuronal factors is investigating the co-localization of these substances with better known neuromediators and/or neuromodulators in the same neuronal cells and nerves. It is known that substances occurring within the same nervous structures most frequently play similar roles [45]. The present study has shown that NRG-1-positive nerves in the porcine uterus may also simultaneously contain the presence of SP, VIP, GAL, DBH, and CART. SP is known as one of the most important sensory factors, and is involved in both sensory and pain stimuli conduction from the reproductive organs to the central nervous system [46]. The other function of SP are stimulatory effects on smooth muscles [47] and participation in immune processes [48]. In turn, VIP is a potent inhibitory factor, which causes vasodilation, relaxation of the smooth muscles, and reduction of mucosal secretory activity [49]. Contrary to SP and VIP, functions of GAL and CART in the uterine innervation are not fully explained. It is assumed that GAL may influence the uterine muscles, but the degree of this impact clearly depends on age, species of animal, and probably on the stage of the estrous cycle [50]. Moreover, the correlation between sex hormones and expression of GAL in the nervous structures supplying the uterus has been investigated, which strongly suggests that the participation of GAL in the regulation of uterine functions depends on the period of the estrous cycle [51]. 

In turn, the functions of CART in the reproductive system still remain unknown, although some previous studies suggest the roles of this peptide in regulating the activity of the smooth muscles of various internal organs [52]. Co-localization of NRG-1 with the above mentioned neuronal active factors observed during the present study strongly suggest that this substance may play similar roles to these factors, but simultaneously multidirectional functions in the uterine innervation. 

The next substance which has been observed in NRG-positive nerves was DBH, used here as a marker of adrenergic innervation. It is known that this innervation plays an important role in the regulation of contractility of the uterus, and the character of these changes depends on the type of activated receptor. Contractions of the uterine smooth muscles have been noted, α-adrenergic receptors are stimulated, while the action of β-adrenergic receptors has shown relaxant effects [53]. Moreover, adrenergic nerves take part in the regulation of blood flow in the uterine wall, and their activation results in vasoconstriction [54]. However, the small number of nerves simultaneously immunoreactive to NRG-1 and DBH, noted during the present study, may suggest that NRG-1 is not an important active substance in the adrenergic innervation of the uterus.

The results obtained during the present study show that low and high doses of BPA may change the number and neurochemical characterization of NRG-1-LI nerves in the uterine wall. These observations suggest that NRG-1 in uterine nerves takes part in pathological processes. It is in accordance with previous investigations, which have described the participation of NRG-1 in mechanisms connected with various diseases, mainly within the gastrointestinal tract. First of all, this substance is probably involved in the pathogenesis of Hirschsprung’s disease, consisting of the deficiency of neuronal structures within the colon [55,56,57]. Besides Hirschsprung’s disease, NRG-1 may also take part in other intestinal pathological states characterized by atrophy of enteric nervous structures, including diverticulosis, slow-transit constipation, or gastrointestinal neuromuscular diseases [55]. In these diseases, NRG-1 plays neuroprotective and/or adaptive functions, which aim to maintain homeostasis and ensure neuronal functioning in conditions changing by pathological processes. Probably similar functions of NRG-1 are at the heart of changes observed during the present study. It is more likely that BPA is a relatively well-known neurotoxic factor which disturbs proper functioning of synapses and development of dendrites and axons [58] through influences on gene expression in the neuronal cells and associated expression of neuronal active substances [23]. The increase of the number of nerves showing the presence of NRG-1, which has a neuroprotective activity [59], may be the answer for these processes. Such a thesis is also supported by the fact that the expression of other neuronal substances (including VIP, GAL, SP, and CART), which co-localize with NRG-1 in the same nerves and which are considered to be important neuroprotective factors [60,61,62] is also higher after BPA administration.

Indeed, changes observed during the present study can also result from not only direct neurotoxic effects of BPA, but also from other mechanisms. One of them may be the pro-inflammatory effects of BPA and its influence on the immunological system [63]. It is known that this substance increases synthesis of pro-inflammatory cytokines [64] and, affecting the estrogen receptors, decreases the number of T and B lymphocytes [65]. Relatively well-known close cooperation between the nervous and immune systems [66] suggests that fluctuations noted in the present study may be connected with these activities. Moreover, all neuronal factors studied in the present investigations in NRG-1-positive nerves are known as substances acting on the immune system. Namely, SP through receptors on lymphocytes and macrophages enhances the secretion of pro-inflammatory factors [67], VIP keeps the balance between pro- and anti-inflammatory cytokines [68,69], and GAL is an anti-inflammatory substance which increases the levels of IFN-γ and IL-12/23 [70]. Even CART, which has roles in the modulation of the immune system, still remains not fully elucidated and plays important functions in modulating post-stroke immune processes [71].

The next reason for observed changes, especially in nerves located in the muscular layer of the uterus, may be connected with the direct impact of BPA on smooth muscles. Previous studies have shown that this substance within muscular tissue affects Maxi-K (KCa.1.1) channels and the guanylyl cyclase (GC) signaling pathway, resulting in the relaxation of muscles [72,73]. On the other hand, NRG-1 is known as a factor which participates in the proper functioning of smooth muscles [74]. Admittedly, previous studies have described such functions of NRG-1, mainly in vascular smooth muscles [75], but it cannot be excluded that similar activity also concerns the uterus. Furthermore, other neuronal factors studied in the present experiment (as mentioned above) play important roles in regulation of the smooth muscular activity [76,77,78,79]. Within the muscular layer, the most interesting was the BPA-induced reaction of nerves simultaneously immunoreactive to NRG 1 and GAL in the uterine horn. The percentage of these nerves was the highest in animals treated with low doses of BPA, while under high doses of the toxin this value was higher than in control animals, but clearly lower than in the E2 group. The reason of such a situation is unknown. It can be only supposed that in this case two mechanisms of action of BPA and two functions of GAL may overlap. It is possible that changes observed in animals under small doses of BPA result first of all from neurotoxic effects of the toxin and reaction of GAL as a neuroprotective and/or anti-inflammatory factor [80]. However, under high doses of BPA, apart from neurotoxic effects, the relaxant action of the toxin may become more important [81], which in turn causes the inhibition of GAL, also known as a substance taking part in the contraction of the smooth muscles [82].

Another cause of the changes noted in the present study may be connected with sensory and pain stimuli conduction, but this thesis is rather controversial. On the one side, the participation of NRG-1 in sensory conduction has been confirmed by previous studies [83]. Not only SP, which seems to be one of the most important sensory factors [84,85], but also other neuronal substances co-localized with NRG-1 in uterine nerves, are known as neuromediators and/or neuromodulators occurring in sensory neurons [36,86,87,88,89]. On the other hand, it should be underlined that doses of BPA used in this investigation were relatively low and should not cause pain signals, despite the pro-inflammatory properties of this substance. Moreover, experimental animals after BPA administration did not manifest pain signals. For these reasons, the thesis that observed changes are connected with pain is rather unlikely.

To sum up, the results obtained during the present study clearly show that NRG-1 participates in uterine innervation. The relatively high number of nerves showing the presence of this substance may suggest its important functions. In turn, the presence of NRG-1-LI fibers in various parts of the uterine wall, as well as a wide range of substances co-localized with NRG-1 in the same nerve fibers, may indicate multidirectional roles of this glycoprotein in nervous structures supplying the reproductive system. Fluctuations in the number of NRG-1-positive nerves and changes in their neurochemical characterization under the impact of BPA confirm the participation of this substance in pathological processes. The observed changes are probably the result of neurotoxic effects of BPA and are connected with known neuroprotective and/or adaptive functions of NRG-1 from previous studies. Nevertheless, observed fluctuations may result from other activities of BPA, including pro-inflammatory properties and/or direct impact on the muscular tissue. Due to the fact that observed fluctuations may be connected with various changes in different stages of protein synthesis, as well as disturbances in intraneuronal transport from perikaryon to neuronal processes, the exact explanation of functions of NRG-1 under the influence of BPA requires further studies.

## 4. Materials and Methods

This study was performed on 15 immature Piétrain × Duroc sows (8 weeks old and about 20 kg body weight), which during the experiment were kept in standard laboratory conditions and fed with complete commercial feed appropriate for age and species of animals. Pigs were kept in pens of an area of about 6 m^2^ (five animals in each pen) and fed using the commercial all-mash feed for piglets “WIGOR 3” (WIPASZ S.A, Olsztyn, Poland). Feed was administered twice a day (in the morning and evening) in quantities of 1 kg/animal/day. Moreover, all animals had free access to water. All activities made during the experiment have obtained the permit from Local Ethical Committee for Experiments on Animals (University of Warmia and Mazury in Olsztyn, Poland) (decisions number 28/2013 of 22 May 2013 and 65/2013/DLZ of 27 November 2013). Animals were randomly divided into three groups consisting of 5 pigs each, according to previously described experimental design [90] on control, experimental I (E1), and experimental II (E2) groups. Control animals received empty capsules (gelatin capsules, Carlson Laboratories, Arlington Heights, IL, USA) for 28 days (per os, once a day, before morning feeding. In the case of E1 group, the capsules contained BPA in a dose 0.05 mg/kg b.w./day, and in the case of E2 group, the dose of BPA was ten times higher (0.5 mg/kg b.w./day). The administration of capsules in E1 and E2 groups was the same as in control animals. Every five days, all animals were weighed to correlate the quantity of BPA in the capsules. After 28 days of BPA administration, all pigs were euthanized. For this purpose, animals were premedicated with Stresnil (Janssen, Belgium, 75 μL/kg of b.w., given intramuscularly) and about 20 min after premedication an overdose of thiopental (Thiopental, Sandoz, Kundl, Austria, given intravenously) was given intravenously. Immediately after euthanasia, the uteri were collected from all animals. Collected organs were fixed in 4% buffered paraformaldehyde (pH 7.4) at room temperature (rt) for 1 h, rinsed in phosphate buffer for three days, and put into 18% phosphate-buffered sucrose solutions. Uteri were storage at this solution for three weeks at 4 °C. Then fragments of uterine corpus (ca. 0.5 cm long, collected just below the uterine bifurcation) and two uterine horns (ca. 0.5 cm, collected about 2 cm above the place where the uterine corpus transitions into horns) were frozen at −22 °C, cut into 14 μm-thick sections with the cryostat (HM 525, Microm International, Germany), and put on microscopic slides. Sections of the uterus were subjected to routine immunofluorescence method describing previously by [90]. This method consisted of drying the slides with the uterine fragments (1 h, room temperature (rt)), treatment of the sections with solution preventing the non-specific labelling composed of 10% normal goat serum, 0.1% bovine serum albumin, 0.01% NaN_3_, Triton x-100, and thimerozal in PBS (humid chamber, 1 h, rt), and incubation with antibodies against neuregulin 1 (NRG-1), dopamine beta-hydroxylase (DBH—used here as a marker of sympathetic nerve fibers), vasoactive intestinal polypeptide (VIP), galanin (GAL) neuronal isoform of nitric oxide synthase (nNOS—a marker of nitrergic nerves) substance P (SP), as well as cocaine- and amphetamine-regulated transcript (CART) (Table 3) (humid chamber, overnight, rt). The next day, complexes “antigen-antibody” were visualized by the incubation of uterine sections with species-specific antisera conjugated with appropriate fluorochromes (humid chamber, 1 h, rt, Table 3). The final stage of the method was the treatment of the uterine fragments with buffered glycerol, and covering them with coverslips. The specification of antibodies using in this study are present in table. During the present study, routine checking of antibodies specificity were performed, such as pre-absorption of each antibody with appropriate antigen, omission, and replacement tests. 

The study of labelled fragments of the uterus were performed with immunofluorescence microscope Olympus BX51 (Japan) fitted with appropriate filter sets. The evaluation of the number of nerves immunoreactive to NRG1 consisted of counting such fibers in the microscopic observation field (0.1 mm^2^). The counting was made up in five randomly selected observation fields located on six uterine sections (evaluation of the number of NRG-1-LI nerves was conducted on 30 microscopic observation fields from each animal). To prevent double counting, the same nerve sections included in the experiment were placed at least 100 µm apart.

The neurochemical coding of fibers immunoreactive to NRG-1 was established by the examination of at least 500 NRG-1-LI fibers located in each part of the uterus from each animal on the simultaneous presence of other neuronal factors studied. These evaluations were conducted on at least ten sections of the uterus for each neuronal substance studied, and the number of NRG-1-LI fibers included into the experiment was considered as representing 100%. The results were pooled and presented as a mean ± SEM. Microphotographs were performed using XM 10 digital camera (Olympus, Japan). Statistical analysis was carried out using Kruskal–Wallis test (Statistica 9.1, StatSoft, Inc., Cracow, Poland). The differences were considered statistically significant at *p* ≤ 0.05.

## Figures and Tables

**Table 1:** Neuregulin-1-positive nerve fibers and their neurochemical characterization in the uterine muscular layer of the control animals (Control) and pigs treated with low (E1) and high (E2) doses of bisphenol A.

Muscular Layer									
Neuronal Factor	Corpus of the Uterus			Right Horn			Left Horn		
	Control	E1	E2	Control	E1	E2	Control	E1	E2
NRG-1^+ 1)^	5.45 ± 0.52 ^a^	8.38 ± 0.43 ^b^	12.29 ± 0.58 ^c^	3.89 ± 0.38 ^a^	5.38 ± 0.35 ^b^	9.2 ± 0.46 ^c^	3.78 ± 0.34 ^a^	5.18 ± 0.35 ^b^	9.97 ± 0.37 ^c^
NRG-1^+^/DBH^+ 2)^	0.34 ± 0.06 ^a^	3.45 ± 0.30 ^b^	8.31 ± 0.39 ^c^	2.77 ± 0.24 ^a^	10.45 ± 0.44 ^b^	11.69 ± 0.12 ^b^	2.85 ± 0.27 ^a^	8.89 ± 0.41 ^b^	11.26 ± 0.20 ^c^
NRG-1^+^/GAL^+ 2)^	14.59 ± 0.97 ^a^	31.38 ± 1.75 ^b^	30.58 ± 1.69 ^b^	10.15 ± 0.85 ^a^	25.79 ± 1.18 ^b^	19.58 ± 1.02^c^	9.62 ± 0.89 ^a^	27.43 ± 1.67 ^b^	20.49 ± 1.36 ^c^
NRG-1^+^/VIP^+ 2)^	31.79 ± 1.21 ^a^	42.28 ± 1.17 ^b^	49.24 ± 1.25 ^c^	26.02 ± 1.13 ^a^	29.74 ± 1.55 ^a^	33.85 ± 1.20 ^b^	26.67 ± 2.20 ^a^	29.04 ± 1.55 ^a^	33.42 ± 1.21 ^b^
NRG-1^+^/nNOS^+ 2)^	25.73 ± 0.98 ^a^	40.10 ± 1.38 ^b^	49.92 ± 1.62 ^c^	20.60 ± 1.25 ^a^	30.49 ± 0.96 ^b^	37.56 ± 0.83 ^c^	21.09 ± 1.04 ^a^	29.96 ± 1.09 ^b^	37.38 ± 1.42 ^c^
NRG-1^+^/SP^+ 2)^	34.37 ± 1.63 ^a^	38.14 ± 2.05 ^a^	39.03 ± 1.73 ^a^	29.15 ± 2.12 ^a^	32.02 ± 0.94 ^a^	34.5 ± 1.38 ^b^	28.97 ± 1.28 ^a^	32.52 ± 1.65 ^a^	34.35 ± 1.21 ^b^
NRG-1^+^/CART^+ 2)^	24.6 ± 1.15 ^a^	30.35 ± 1.46 ^b^	42.49 ± 1.48 ^c^	19.02 ± 1.08 ^a^	26.45 ± 1.63 ^b^	39.34 ± 1.93 ^c^	18.6 ± 1.72 ^a^	25.12 ± 1.27 ^b^	39.23 ± 1.78 ^c^

NRG-1—neuregulin 1, DBH—dopamine beta-hydroxylase (used here as a marker of sympathetic nerve fibers), GAL—galanin, VIP—vasoactive intestinal polypeptide, nNOS—neuronal isoform of nitric oxide synthase (a marker of nitrergic nerves), SP—substance P, CART—cocaine- and amphetamine-regulated transcript. ^1)^ The average number of fibers in the microscopic observation field (0.1 mm^2^); ^2)^ the percentage of nerves immunoreactive to the particular substances in respect to all NRG-1-positive nerves (NRG-1-positive nerves were considered as representing 100%). Statistically significant data (*p* ≤ 0.05) in particular rows are marked by different letters, and not significant data are marked by the same letters.

**Table 2:** The number of Neuregulin-1-positive fibers and their neurochemical characterization in the uterine mucosal layer of the control animals (Control) and pigs treated with low (E1) and high (E2) doses of bisphenol A.

Mucosal Layer									
Neuronal Factor	Corpus of the Uterus			Right Horn			Left Horn		
	Control	E1	E2	Control	E1	E2	Control	E1	E2
NRG-1^+ 1)^	5.08 ± 0.43 ^a^	4.78 ± 0.43 ^a^	7.86 ± 0.29 ^b^	2.93 ± 0.45 ^a^	3.19 ± 0.31 ^a^	5.7 ± 0.54 ^b^	3.08 ± 0.32 ^a^	3.47 ± 0.44 ^a^	6.25 ± 0.45 ^b^
NRG-1^+^/DBH^+ 2)^	1.87 ± 0.41 ^a^	10.73 ± 0.57 ^b^	17.35 ± 1.08 ^c^	7.32 ± 0.62 ^a^	19.97 ± 0.87 ^b^	38.62 ± 1.73 ^c^	7.26 ± 0.99 ^a^	20.71 ± 1.36 ^b^	39.76 ± 1.06 ^c^
NRG-1^+^/GAL^+ 2)^	10.52 ± 0.47 ^a^	25.86 ± 1.04 ^b^	25.27 ± 0.7 ^b^	7.99 ± 0.77 ^a^	21.76 ± 0.7 ^b^	20.00 ± 1.17 ^b^	8.15 ± 0.79 ^a^	21.58 ± 1.24 ^b^	19.84 ± 1.06 ^b^
NRG-1^+^/VIP^+ 2)^	24.1 ± 0.98 ^a^	35.53 ± 0.69 ^b^	41.44 ± 0.97 ^c^	21.02 ± 1.46 ^a^	24.83 ± 0.95 ^a^	24.5 ± 1.24 ^a^	21.96 ± 1.03 ^a^	24.51 ± 1.93 ^a^	23.74 ± 1.03 ^a^
NRG-1^+^/nNOS^+ 2)^	22.30 ± 1.06 ^a^	39.13 ± 1.72 ^b^	45.33 ± 1.99 ^c^	15.85 ± 0.85 ^a^	26.15 ± 0.89 ^b^	34.44 ± 1.54 ^c^	16.07 ± 0.74 ^a^	26.74 ± 1.55 ^b^	34.56 ± 2.19 ^c^
NRG-1^+^/SP^+ 2)^	31.55 ± 0.92 ^a^	34.25 ± 0.76 ^b^	34.05 ± 1.75 ^b^	30.45 ± 0.78 ^a^	35.41 ± 0.99 ^b^	32.24 ± 1.07 ^a^	30.72 ± 1.15 ^a^	34.89 ± 1.72 ^b^	33.02 ± 1.30 ^a^
NRG-1^+^/CART^+ 2)^	18.56 ± 1.39 ^a^	22.61 ± 0.82 ^a^	36.36 ± 1.67 ^b^	14.77 ± 1.13 ^a^	18.86 ± 1.25 ^a^	36.35 ± 0.99 ^b^	14.3 ± 1.08 ^a^	18.46 ± 1.85 ^a^	35.59 ± 1.43 ^b^

NRG-1—neuregulin 1, DBH—dopamine beta-hydroxylase (used here as a marker of sympathetic nerve fibers), GAL—galanin, VIP—vasoactive intestinal polypeptide, nNOS—neuronal isoform of nitric oxide synthase (a marker of nitrergic nerves), SP—substance P, CART—cocaine- and amphetamine-regulated transcript. ^1)^ the average number of fibers in the microscopic observation field (0.1 mm^2^); ^2)^ the percentage of nerves immunoreactive to the particular substances in respect to all NRG-1-positive nerves (NRG-1-positive nerves were considered as representing 100%). Statistically significant data (*p* ≤ 0.05) in particular rows are marked by different letters, and not significant data are marked by the same letters.

**Table 3:** Description of antibodies.

Antigen	Species of Origin	Code	Supplier
**Primary Antibodies**			
GAL	Guinea pig	T-5036	PENINSULA
nNOS	Mouse	N218	Sigma-Aldrich
VIP	Mause	9535-0504	BIOGENE
SP	Rat	8450-0505	AbDserotec
CART	Mouse	R&D	MAB 163
Neu-1	Rabbit	AA 198-229	antibodies-online
DBH	Mouse	MAB308	Chemicon
**Secondary Antibodies**			
Alexa fluor 488	Anti Rabbit	A21206	Invitrogen
Alexa fluor 546	Anti Mouse	A10036	Invitrogen
Alexa fluor 546	Anti Rat	A11081	Invitrogen

## Abbreviations

BPA	bisphenol A
NRG-1	neuregulin 1
GAL	galanin
nNOS	neuronal isoform of nitric oxide synthase
VIP	vasoactive intestinal polypeptide
SP	substance P
CART	cocaine- and amphetamine-regulated transcript
DBH	dopamine beta-hydroxylase
